# Research on Lateral Safety Spacing for Fusion Operation Based on Unmanned and Manned Aircraft-Event Modeling

**DOI:** 10.3390/s24020553

**Published:** 2024-01-16

**Authors:** Chao Zhou, Chi Huang, Longyang Huang, Chuanjiang Xie, Xingyu Zhu, Tao Huang

**Affiliations:** 1 Institute of Electronic and Electrical Engineering, Civil Aviation Flight University of China, Guanghan 618307, China; 18428327587@163.com (C.X.); 15520664026@163.com (T.H.); 2 College of Air Traffic Management, Civil Aviation Flight University of China, Guanghan 618307, China; hc13778880943@163.com (C.H.); longyanghuang@cafuc.edu.cn (L.H.); zhcnzxy@gmail.com (X.Z.)

**Keywords:** manned and unmanned aircraft, collision risk, safety space

## Abstract

With the rapid development of unmanned aerial vehicle technology and its increasing application across various fields, current airspace resources are insufficient for unmanned aerial vehicles’ needs. This paper, taking Zigong General Aviation Airport in Sichuan as a case study, explores the lateral safety spacing in a mixed operation mode of unmanned aerial vehicles and manned aircraft. Currently, there are no standardized regulations for the safe spacing of the fusion operation of unmanned and manned aircraft. Theoretical research is essential to provide a reference for actual operations. It introduces the UM-Event (unmanned and manned aircraft-event) collision risk model, an adaptation of the Event collision risk model, considering factors like communication, navigation, surveillance performance, human factors, collision avoidance equipment performance, and meteorology. Safety spacing was determined via simulation experiments and actual data analysis, adhering to the target safety level (TSL). Findings indicate that surveillance performance has a minor impact on safety spacing, while communication and navigation significantly influence it. The safety spacing, influenced solely by CNS (communication performance, navigation performance, surveillance performance) and combined factors, increased from 4.42 to 4.47 nautical miles. These results offer theoretical guidance for unmanned aerial vehicle safety in non-segregated airspace.

## 1. Introduction

Recently, low-altitude airspace has experienced rapid development, with drones flourishing and artificial intelligence significantly advancing the drone market [1,2]. Concurrently, advancements in UAV communication [3], line-of-sight and over-the-horizon navigation [4], surveillance [5], mission payloads, and cruise inspection technologies are notable [6]. In this context, the demand for UAVs for airspace resources is increasing, and there is an urgent need to make full use of existing airspace resources in order to improve the efficiency of airspace utilization and maximize the value of airspace resources. Therefore, making full use of airspace resources and realizing the fusion operation of manned aircraft and UAVs [7] have become the future development direction, and solving the risk of collision between UAVs and manned aircraft [8] and the safety spacing problem is also becoming increasingly important. Given these advancements, the demand for airspace resources by UAVs is escalating, necessitating the efficient utilization and maximization of existing airspace resources [9]. Consequently, fully leveraging these resources to integrate manned and unmanned aircraft operations is emerging as a key future trend. Addressing the collision risks and safety intervals between unmanned and manned aircraft is becoming increasingly crucial [10].

Reich et al. introduced the Reich model for parallel flight paths, focusing on aircraft position, velocity, and random deviation [11]. Subsequently, Prof. Brooker from the UK developed the EVENT collision risk model, advancing the Reich model to assess minimum safe aircraft spacing. This model offers innovative insights and theoretical support for aircraft flight safety analysis [12,13]. Lili Wang and colleagues designed a collision risk assessment model for small UAVs in low-altitude airspace, accounting for different flight maneuvers and the random speed distribution of these UAVs, to understand the correlation between collision risk and UAV density [14]. In the context of mixed operations of UAVs and manned aircraft, Qing Yuan Yu and team categorized UAVs by risk altitude layers, calculating the collision control intervals between UAVs and manned aircraft, considering command delays [15]. Tamer Savas and associates proposed a new UAV path model for integrating UAVs into non-segregated airspace, validated using real-time simulation [16]. In 2022, Noh S and John Shortle introduced the Dynamic Event Tree (DET) framework for assessing collision risks and safety spacing in various aircraft, applying it to simulated collision scenarios [17]. By 2023, Figuet B had developed a novel, data-driven method using Monte Carlo simulation and extreme value theory to estimate in-air collision probabilities, offering significant improvements over traditional approaches for assessing aircraft collision risk and safety spacing [18].

With the current deepening of the research, domestic and international research on the collision risk factors between aircraft focuses on communication, navigation, and surveillance performance (CNS performance) [19]. To study the effect of CNS performance on aircraft positioning error, we establish a multi-aircraft collision risk model, analyze the collision risk under different spacing by arithmetic examples, and determine the minimum safe spacing, which is practical and scalable for the development of UAV traffic management and network design lateral isolation standards [20]. This paper is currently selected to systematically analyze them from the perspective of communication, navigation, and surveillance performance [21]. At the same time, there are some researchers who joined this with the study of human factors. Kelly D [22] introduced the analysis of human factors into 50 controlled flights concerning ground aviation accidents from 2007 to 2017, which comprised different perspectives, qualitative dimensions of the human factor, quantitative elaboration, and human causes that contribute to the risk of collision between the aircraft due to equipment factors. The causes of human-caused collision risk include navigational errors due to equipment factors that cause the aircraft to deviate from the designated flight path and flight deviations due to subjective human errors that cause the aircraft to fly on a non-designated flight path. However, fusion operation implies the safe fusion operation of unmanned and manned aircraft, and factors affecting safety must be considered in various aspects, and it has also been shown that onboard collision avoidance equipment (TCAS) and meteorological conditions have a greater impact on fusion operation. The study by Zhaoning Zhang [23] shows that onboard collision avoidance equipment, as a vital safety measure for the fusion operation of unmanned and manned aircraft, plays a key role in ensuring the safety and synergy of aviation activities. Schäfer M [24] and Xiaohan Liao et al. [25] described that the onboard equipment of an aircraft draws on a variety of advanced technologies, such as radar, cameras, and infrared sensors, in order to monitor the environment around the aircraft in real time, to warn in advance, and to avoid potential collision risks [26], not to mention the meteorological conditions, whose wind speed, temperature, and barometric pressure all have a greater impact on the aircraft. Qi Li [27] and others described how aircraft flights are usually affected by high-altitude winds, and that different wind directions lead to different trajectories, and added meteorological factors to the modeling calculations [28]. Different meteorological conditions can lead to changes in the refractive index of the atmosphere, which affects the speed and direction of signal transmission, and thus the positioning accuracy. In a positioning system, the speed and direction of signal transmission are very important. When the signal passes through atmospheric layers of different densities, refraction occurs, leading to changes in the speed and direction of signal transmission. This change causes the signal to arrive at the receiver at a different time and in a different direction than expected, resulting in aircraft positioning errors [29].

Therefore, in-depth studies on collision risk and safe spacing of UAVs in converged airspace are needed in order to predict the conflict threat to manned aircraft in a timely manner [30]. The above studies demonstrated the research progress for aircraft collision risk and safe spacing, involving the application of different models and methods, as well as the analysis of collision risk between UAVs and manned aircraft, which are important to ensure the safety of aircraft in flight [31]. However, the above studies are not comprehensive enough to consider the safety factors affecting the fusion operation of manned and unmanned aircraft, and in this paper, we will comprehensively consider the influence of multiple factors on the lateral safety spacing of fusion operation [32]. Firstly, we will introduce the domestic and international research on the collision risk of fusion operation; analyze the influence of CNS performance, human factors, TCAS equipment, and meteorological conditions on the positioning error of the manned and unmanned aircraft in fusion operation; establish the collision risk model of fusion operation according to the influencing factors, mainly the study of the lateral safety spacing of fusion operation between the unmanned aircraft and the manned aircraft; and use the algorithm to calculate the lateral safety spacing of fusion operation. Finally, it is brought into the actual operation scene for simulation verification to analyze the relationship between collision risk and safety spacing.

## 2. Positioning Error Analysis

### 2.1. Errors in CNS Performance

This study’s UAV and manned aircraft flight activities are conducted under performance-based navigation. The positional accuracy error primarily hinges on the CNS performance parameters, encompassing three components: RNP (Required Navigation Performance), RCP (Required Communication Performance), and RSP (Required Surveillance Performance). 

The specific values for these parameters are available in Document 9613 [33], issued by the International Civil Aviation Organization (ICAO).

Let us denote “a” as the parameter value for RNP. $\sigma_{a}$ represents positioning errors due to navigation performance.

Based on the definition of RNP “a” in Table 1, RNP signifies that there is a 95% probability that an aircraft’s navigation accuracy will be within “a” nautical mile of its designated flight path [34]. From this, we can derive the following definition:

(1) $$\int_{-a}^{a}\frac{1}{\sqrt{2\pi \sigma_{a}}}e^{-\frac{x^{2}}{2\sigma_{a}^{2}}}dx\geq 0.95$$

Let “b” represent the parameter value for RCP; “$\sigma_{b}$” stands for positioning error due to communication performance; and “v” is the component of the cruising speed of the aircraft on the line connecting the two aircraft.

According to Table 2, this parameter reflects the maximum processing time, completed in 95% of instances, and is recognized as the operationally acceptable performance, as evidenced by controllers and pilots [35]. Consequently, we deduce the following:

(2) $$\int_{-bv}^{bv}\frac{1}{\sqrt{2\pi \sigma_{b}}}e^{-\frac{x^{2}}{2\sigma_{b}^{2}}}dx\geq 0.95$$

Let “c” be the parameter value for RSP. $\sigma_{c}$ represents the localization error due to the surveillance performance, and “v” is the component of the cruise speed of the aircraft on the line connecting the two aircraft.

As outlined in Table 3, the accuracy of RSP is determined by the radius of the circle around the target location, indicating a 95% probability that the actual target location falls within this circle. From this, we derive the following conclusion:

(3) $$\int_{-cv}^{cv}\frac{1}{\sqrt{2\pi \sigma_{c}}}e^{-\frac{x^{2}}{2\sigma_{c}^{2}}}dx\geq 0.95$$

The following can be found by using the above Equations (1)–(3):

(4) $$\begin{array}{l} \sigma_{a}=\sigma_{n}=a/1.96=0.5102a\\ \sigma_{b}=\sigma_{c}=bv/1.96=0.5102bv\\ \sigma_{c}=\sigma_{s}=cv/1.96=0.5102bv \end{array}$$

Therefore, by Equation (4), it follows that

(5) $$\sigma =\sqrt{\sigma_{n}^{2}+\sigma_{c}^{2}+\sigma_{s}^{2}}=\sqrt{0.2603(a^{2}+b^{2}v^{2}+c^{2}v^{2})}$$

where “v” is the component of the cruising speed of the aircraft on the line connecting the two aircraft; and $\sigma_{n}$, $\sigma_{c}$, and $\sigma_{s}$ denote the localization errors due to navigation, communication, and surveillance performance, respectively. “a”, “b”, and “c” are the values of the RNP, RCP, and RSP parameters, respectively. 

**Figure 1:**
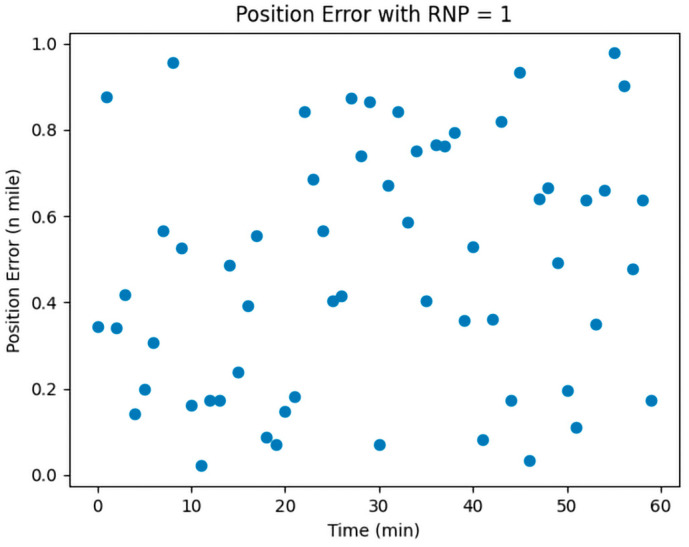
RNP1 error simulation.

**Figure 2:**
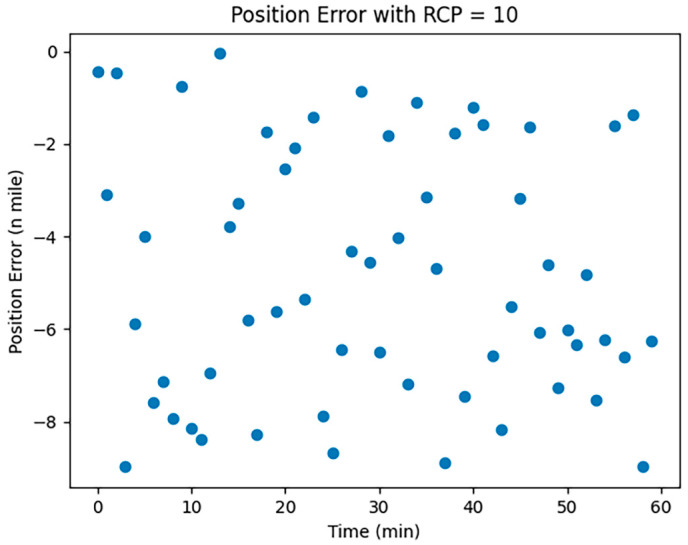
RCP10 error simulation.

**Figure 3:**
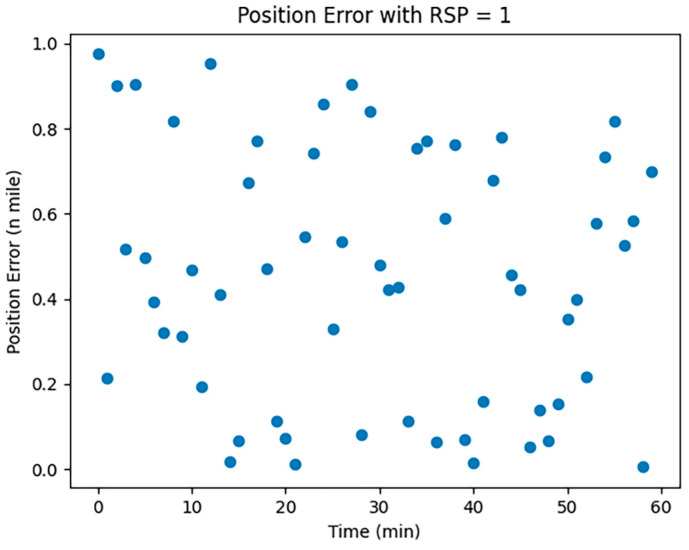
RSP1 error simulation.

CNS was employed to assess the localization errors induced by both UAVs and manned aircraft. Considering the specific conditions of communication, navigation, and surveillance equipment at Zigong Airport, we opted for RNP1, RCP10, and RSP1 to visualize and analyze the errors; the results are shown in Figure 1, Figure 2 and Figure 3:

In Figure 1, Figure 2 and Figure 3, the horizontal coordinates represent the time required in minutes, and the vertical coordinates represent the position error in nautical miles.

The Required Navigation Performance (RNP) is set at a value of 1. Figure 1 illustrates the simulated effect of localization errors, which, over time, show significant instability and randomness without accumulating. This is attributed to RNP’s utilization of advanced navigation equipment and techniques, enabling real-time position monitoring and error correction, thus preventing error accumulation. RNP employs GPS, inertial navigation, and ground radar for multiple position corrections to ensure route accuracy. 

The Required Communication Performance (RCP), with a value of 10, is depicted in Figure 2. RCP-related localization errors tend to be unstable and random, lacking cumulative effect and are influenced by factors like communication disruptions, course deviations, speed changes, timing, and program errors.

The Required Surveillance Performance (RSP), valued at 1, is shown in Figure 3. Similarly, RSP-induced positioning errors display instability and randomness over time without accumulating. These errors may arise from monitoring signals, course deviations, speed changes, timing errors, and program glitches. 

The simulation results indicate that CNS performance significantly impacts aircraft positioning errors, crucially affecting the minimum safe spacing in UAV-manned aircraft fusion operations.

### 2.2. Errors in Other Factors

The horizontal positioning error between aircraft during TCAS conflict resolution can be calculated using the following equation:

(6) $$\varepsilon_{TH}=\varepsilon_{\theta }\cdot d$$

where $\varepsilon_{TH}$ denotes the horizontal positioning error; d is the horizontal distance between two aircraft; and $\varepsilon_{\theta }$ indicates the trajectory angle error, which refers to the deviation between the aircraft’s actual trajectory and the theoretical trajectory, and can be calculated using the following formula: trajectory angle error is equal to the difference between the actual trajectory angle and the theoretical trajectory angle.

The actual trajectory angle is the angle between the actual direction of flight of the aircraft and due north, and can be expressed in mathematical notation as $\theta_{a}$; the theoretical track angle is the angle between the direction in which the aircraft should be flying and due north, and can be expressed in mathematical notation as $\theta_{t}$.

Therefore, the trajectory angle error can be expressed by the following equation:

(7) $$\varepsilon_{\theta }=\theta_{a}-\theta_{t}$$

The horizontal positioning error can be expressed by the following equation:

(8) $$\varepsilon_{TH}=(\theta_{a}-\theta_{t})\cdot d$$

Aircraft positioning errors caused by the influence of human factors (including land and air call delays and human cognitive reliability) can be expressed by the following mathematical formula:

(9) $$\varepsilon_{p}=\varepsilon_{c}+\varepsilon_{h}+\varepsilon_{t}$$

where $\varepsilon_{p}$, $\varepsilon_{c}$, $\varepsilon_{h}$, and $\varepsilon_{t}$ denote the errors due to aircraft localization errors, errors due to land–air call delays, errors due to human cognitive reliability, and errors due to other factors, respectively.

Specifically, the error due to land and air call delays can be calculated using the following formula:

(10) $$\varepsilon_{c}=vt$$

where v and t denote the speed of aircraft operation and the time of land–air call delay, respectively.

The error due to human cognitive reliability can be calculated using the following equation:

(11) $$\varepsilon_{h}=k_{h}\cdot \left(1-p_{h}\right)$$

where $k_{h}$ denotes a constant to regulate the effect of human cognitive reliability on the error, and $p_{h}$ denotes human cognitive reliability and takes values ranging from 0 to 1.

The error due to other factors can be calculated using the following formula:

(12) $$\varepsilon_{t}=k_{t}\cdot \left(d+v+a\right)$$

where $k_{t}$, d, v, and a denote constants used to regulate the effect of other factors on the error, distance error, velocity error, and indicated acceleration error, respectively.

In summary, the horizontal positioning error of an aircraft caused by human factors can be expressed as

(13) $$\varepsilon_{p}=vt+k_{h}\cdot \left(1-p_{h}\right)+k_{t}\cdot \left(d+v+a\right)$$

The impact of meteorological conditions on positioning errors is multifaceted, involving various factors. Predominantly, changes in the atmospheric refractive index significantly affect positioning accuracy by altering the speed and direction of signal transmission. These changes are influenced by atmospheric conditions like temperature, humidity, and pressure. The corresponding atmospheric refraction error can be quantified using a specific formula, which takes into account these meteorological parameters:

(14) $$\varepsilon_{Ari}=\int n-1ds$$

where $\varepsilon_{Ari}$ denotes the atmospheric refractive index error, n is the atmospheric refractive index, and s is the distance on the flight path of the aircraft. Since the atmospheric refractive index is related to temperature, humidity, and other factors, it is necessary to consider the influence of these factors on the atmospheric refractive index.

The atmospheric refractive index can be calculated using the following formula:

(15) $$\begin{array}{l} n=1+77.6\times 10^{-6}P-5.6\times 10^{-7}T+(3.73\times 10^{-6}P-0.042T-0.0029H)\\ \times 10^{-6}(\lambda^{2}+0.011\lambda +0.0003) \end{array}$$

where P is the atmospheric pressure, T is the temperature, H is the relative humidity, and λ is the wavelength of light.

Meteorological conditions can impact the distance and strength of signal transmission, consequently affecting positioning accuracy. These variations can be measured by factors like atmospheric transparency and water vapor content. The signal transmission error, which reflects the impact of these variations on an aircraft’s positioning error, can be calculated using a specific formula that considers these meteorological influences:

(16) $$\varepsilon_{ST}=10\log\frac{P_{t}}{P_{r}}+20\log d+K$$

where $\varepsilon_{ST}$ denotes the signal transmission error, $P_{t}$ is the transmit power, $P_{r}$ is the receive power, d is the signal transmission distance, and K is a constant.

The transmit power and receive power can be calculated using the following equations:

(17) $$P_{t}=G_{t}P$$

(18) $$P_{r}=G_{r}P_{t}{\left(\frac{\lambda }{4\pi d}\right)}^{2}$$

where $G_{t}$ is the transmit antenna gain, P is the transmitter output power, $G_{r}$ is the receive antenna gain, and λ is the signal wavelength. Variations in signal transmission distance and strength may be affected by a variety of factors, such as weather, terrain, obstacles, etc. Therefore, K is a constant to account for the effect of these factors on signal transmission.

The thickness and density of the atmosphere influence the signal’s transmission path and speed, subsequently affecting positioning accuracy. These atmospheric properties are determined by factors like temperature and pressure. The atmospheric error, which accounts for the impact of atmospheric thickness and density on aircraft positioning errors, can be calculated using the following formula:

(19) $$\varepsilon_{ATM}=0.5C_{d}\rho V^{2}S$$

where $\varepsilon_{ATM}$ denotes the atmospheric error, $C_{d}$ is the drag coefficient of the aircraft, ρ is the atmospheric density, V is the speed of the aircraft, and S is the reference area of the aircraft.

Atmospheric density can be calculated using the following formula:

(20) $$\rho =\rho_{0}e^{-\frac{h}{H}}$$

where $\rho_{0}$ is the atmospheric density at sea level, h is the altitude of the aircraft, and H is the thickness of the atmosphere.

Effect of wind: Wind in the air can cause an aircraft to deviate from its intended trajectory, resulting in a positioning error. Wind error is the effect of wind on the positioning error of an aircraft and can be calculated using the following formula:

(21) $$\varepsilon_{W}=V_{W}\sin\alpha$$

where $V_{W}$ is the wind speed, and α is the angle between the wind direction and the heading.

The aforementioned factors influencing positioning accuracy can be analyzed and quantified using meteorological observation data and measurements from positioning systems. Understanding the extent of meteorological conditions’ impact on positioning errors allows for the development of targeted measures to enhance positioning accuracy.

Positioning errors caused by meteorological conditions can be synthesized as follows:

(22) $$\varepsilon_{WC}=\varepsilon_{Ari}+\varepsilon_{ST}+\varepsilon_{ATM}+\varepsilon_{w}$$

The error ε caused by the above factors all satisfy the normal distribution law and have randomness; therefore, it is the positioning error caused by UAVs and manned aircraft. We carried out the visual analysis of the error, and the results are shown below.

**Figure 4:**
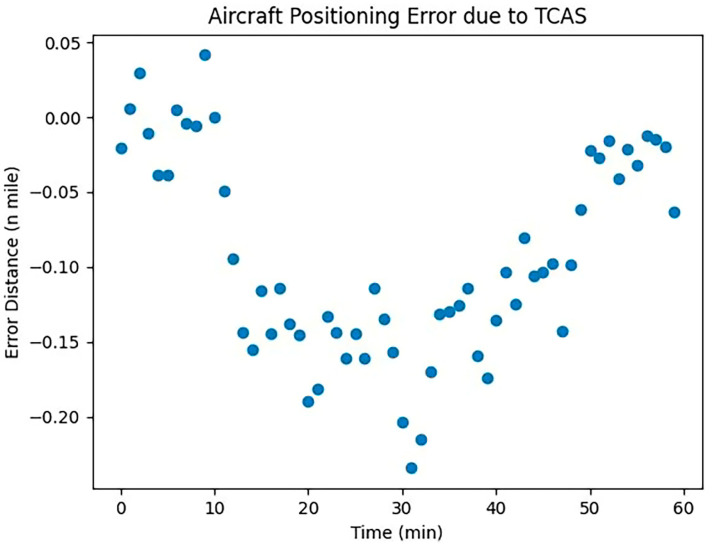
TCAS error simulation.

**Figure 5:**
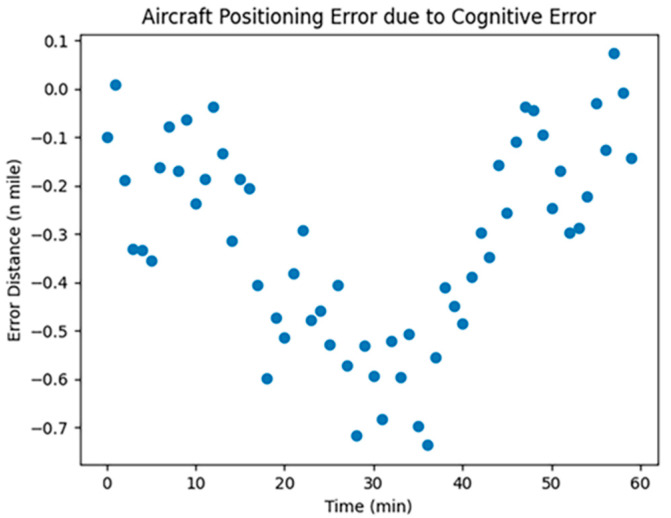
Human error simulation.

**Figure 6:**
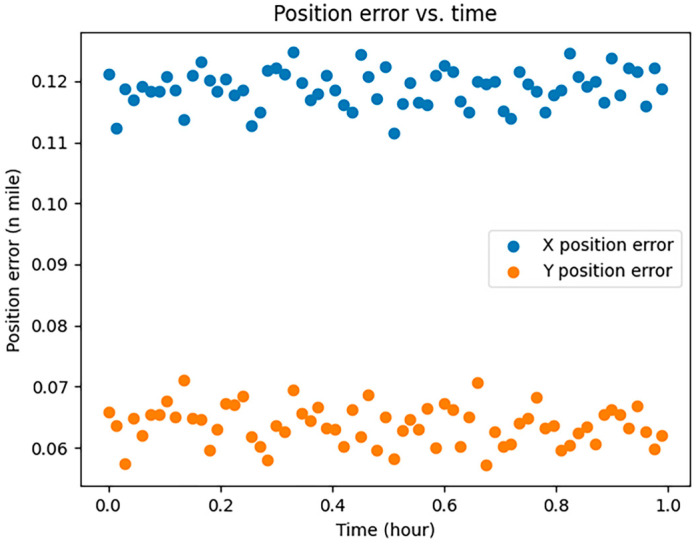
Position errors.

Data from Figure 4, Figure 5 and Figure 6 indicate that the positioning error attributable to collision avoidance equipment gradually increases over time. This trend highlights the varying impact of equipment quality on aircraft, underscoring the need for systematic analysis in future research. Positioning errors due to human factors exhibit significant randomness, reflecting the inherent uncertainties in human cognitive reliability and their influence on aircraft positioning. This randomness mirrors real-world scenarios. Conversely, positioning errors caused by meteorological conditions display a stable pattern consistent with empirical observations. Given the substantial impact of meteorological conditions on aircraft in flight, they warrant focused attention in subsequent research.

## 3. Modeling Crash Risk Calculations

### 3.1. Introduction to the Traditional Event Model

**Figure 7:**
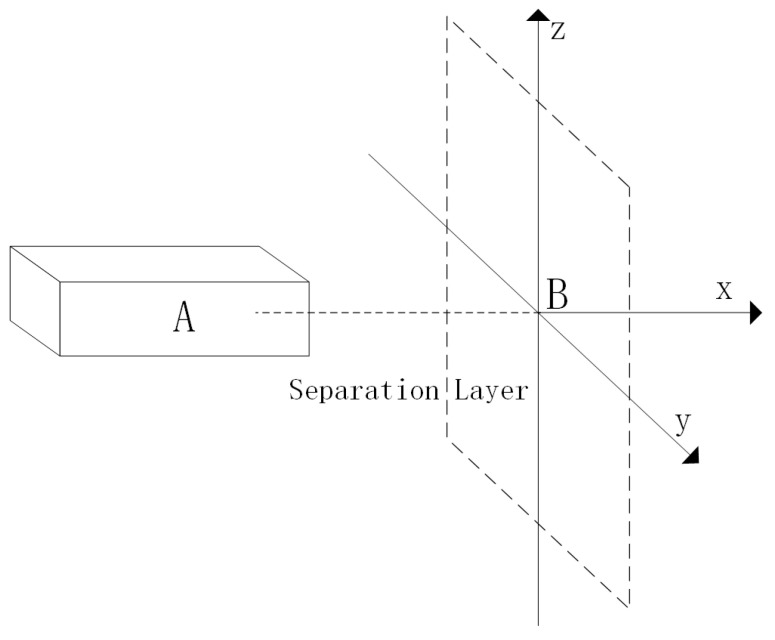
Event crash risk model.

In the Event collision risk model, a rectangular body, labeled as “A”, represents the geometric model of the UAV. This model defines the UAV’s dimensions, where the length, width, and height of the rectangle correspond to the UAV’s length, wingspan, and fuselage height, respectively. Centered around the geometric center of the manned aircraft, a spatial orthogonal coordinate system is established. This system’s x, y, and z axes represent the longitudinal, lateral, and vertical distances, respectively, in the relative motion between the aircraft [36]. A detailed schematic is depicted in Figure 7.

**Figure 8:**
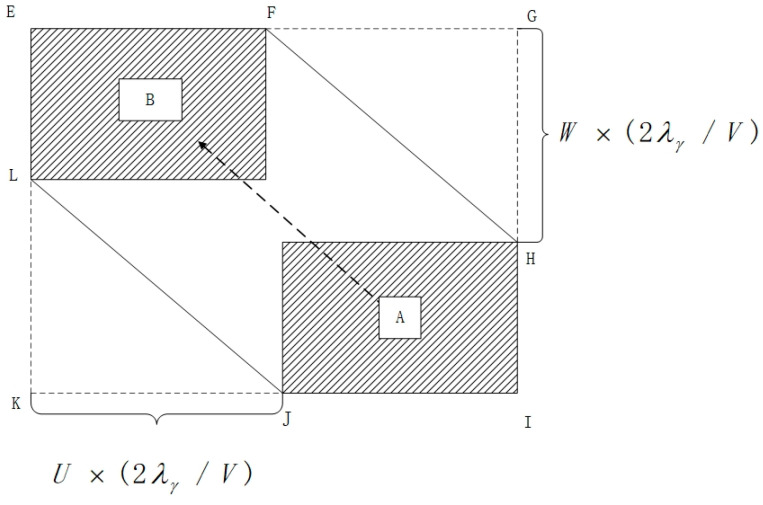
Expanded crash box.

The rectangle EGIK is defined as an extended collision box. A collision is deemed to occur between the manned aircraft (A) and the UAV (B) if the manned aircraft is precisely positioned within rectangle A. This scenario is illustrated in Figure 8. 

In the diagram, “A” represents a manned aircraft, and “B” is an unmanned aircraft. The likelihood of a lateral collision between these two aircraft is determined by multiplying two probabilities: the chance that the manned aircraft “A” is positioned within the extended collision box and the likelihood that the unmanned aircraft “B” is crossing the spacer layer laterally.

(23) $$N_{y}=G_{y}E(0)P_{z}(0)\frac{\lambda_{x}}{S_{x}}(1+\frac{2v_{x}\lambda_{y}}{2v_{y}\lambda_{x}})(1+\frac{2v_{z}\lambda_{y}}{2v_{y}\lambda_{z}})$$

(24) $$P_{y}(S_{y})=2G_{y}\frac{\lambda_{y}}{v_{y}}$$

where

$N_{y}$ is the side impact risk;
E(0) is the longitudinal proximity rate;
$G_{y}$ is the lateral interval loss rate;
$P_{z}(0)$ is the vertical collision probability;
$P_{y}(S_{y})$ is the lateral overlap probability of the two aircraft when the lateral minimum safe spacing is S;
$\lambda_{x}$, $\lambda_{y}$, and $\lambda_{z}$ are the length, wingspan, and height of UAV A, respectively;
$v_{x}$,$v_{y}$, and $v_{z}$ are the relative speeds of the two machines in the longitudinal, lateral, and vertical directions, respectively.

### 3.2. UM-Event Model

Under the influence of CNS localization error, the actual distance between the two aircraft is as follows:

(25) $$D_{1}=d_{1}-X_{1}+X_{2}$$

**Figure 9:**
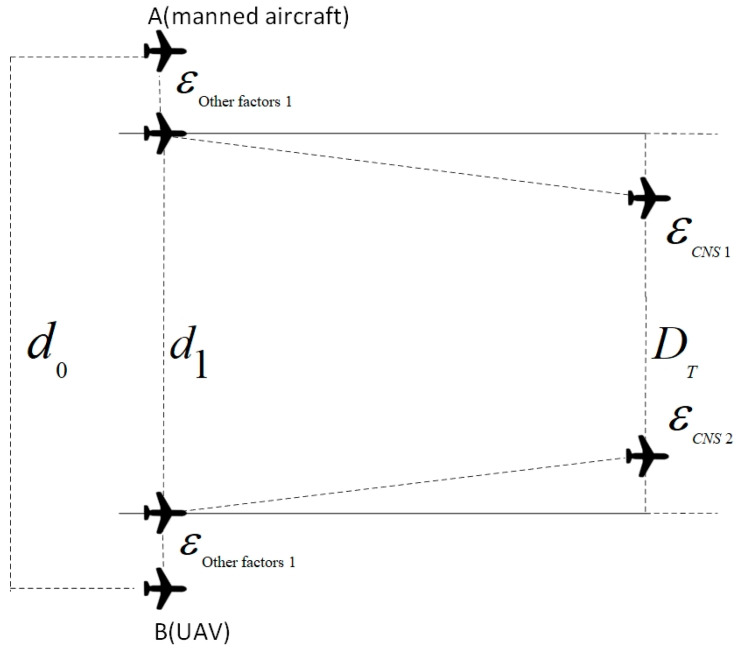
UM-Event model.

Additional factors like onboard collision avoidance equipment, human elements, and meteorological conditions significantly influence the aircraft positioning errors, thereby affecting the actual distance between aircraft. When calculating this distance, it is crucial to incorporate the impact of these variables into the computational model. By utilizing suitable models and algorithms, we can determine how each positioning error and the initial aircraft distance influence the actual distance between aircraft. This approach aims to minimize the safe distance during fusion operation, enhancing flight safety. Figure 9 depicts the relationship between the actual aircraft distance, each localization error, and the initial aircraft distance. 

In Figure 9, we assume that in the absence of external factors such as human errors, onboard collision avoidance systems, and meteorological conditions, the initial positions of the two aircraft are at points A and B, with a lateral distance denoted as $d_{0}$. When these factors are considered, localization errors alter the lateral distance between the aircraft, expressed as $d_{1}$. The calculation process adjusts the aircraft’s initial position to account for the increased impact of positioning errors from various factors. $D_{T}$ in the figure represents the actual distance between the aircraft, factoring in these positioning errors. It is important to note that the figure illustrates an extreme scenario where all positioning errors contribute to a decreased distance between the aircraft, thereby heightening the risk of collision.

At this point, the true distance between the two aircraft can be calculated using Equation (4). (The rightward positioning error of the aircraft is taken as “+”, and the leftward positioning error is taken as “−”):

(26) $$D_{T}=d_{1}-\varepsilon_{CNS1}+\varepsilon_{CNS2}$$

(27) $$d_{1}=d_{0}-\varepsilon_{\mathrm{other}1}+\varepsilon_{\mathrm{other}2}$$

(28) $$\varepsilon_{\mathrm{other}}=\sqrt{\varepsilon_{TH}^{2}+\varepsilon_{P}^{2}+\varepsilon_{WC}^{2}}$$

$\varepsilon_{CNS}$, $\varepsilon_{TH}$, $\varepsilon_{P}$, and $\varepsilon_{WC}$ are the CNS performance error, collision avoidance equipment error, human factor error, and meteorological condition error of the UAV-manned aircraft, respectively. 

Assuming that $d_{0}$ is the initial distance between the two aircraft then at this point, $D_{T}$ satisfies a normal distribution in both directions A and B.

(29) $$D_{T}∼d_{0}+N(0,\sigma_{1}^{2}+\sigma_{2}^{2})$$

(30) $$D_{T}∼N(d_{0},\sigma_{1}^{2}+\sigma_{2}^{2})$$

At this point, the probability density distribution function of the actual distance $D_{T}$ of the aircraft can be expressed as

(31) $${f(D}_{T})=\frac{1}{\sqrt{2\pi (\sigma_{1}^{2}+\sigma_{2}^{2})}}e^{\left[-\frac{{\left(D_{T}-d_{0}\right)}^{2}}{2(\sigma_{1}^{2}+\sigma_{2}^{2})}\right]}$$

Then, the lateral overlap probability P of the two aircraft can be expressed as $L_{1}$ and $L_{2}$, denoting the maximum wingspan of the two aircraft:

(32) $$P_{D_{T}}=\int_{L_{2}}^{L_{1}}f(D_{T})dD_{T}$$

Based on the above analysis, the lateral collision risk model of two aircraft, after considering multiple influencing factors, can be derived as follows:

(33) $$N_{y}=2P_{D_{T}}\cdot E(0)\cdot P_{z}(0)\cdot P_{UM-\mathrm{Event}}$$

where $N_{y}$ is the sideways collision risk probability, and 2 represents two aircraft, manned and unmanned; E(0) $P_{z}(0)$ is the longitudinal proximity rate, and the vertical collision probability, which is not calculated here to take 0 for the time being. $P_{UM-Event}$ denotes the probability of a collision box crossing the spacer layer laterally in the EVENT model. It is directly related to the flight speed of the aircraft and the size of the aircraft.

## 4. Example Calculations

### 4.1. Calculation of Safety Spacing

The collision risk model developed is a nonlinear function, and the Adam iterative algorithm is applied to determine the aircraft’s minimum safe spacing. This algorithm operates by estimating the gradient’s first-order and second-order moments using an exponentially weighted moving average, followed by parameter updates based on these estimates [32]. The detailed procedure is outlined below:

(34) $$f(D)=N_{y}$$

It is translated into an equivalent iterative format:

(35) $$D_{n+1}=D_{n}-\alpha \cdot \frac{m_{n}}{\sqrt{v_{n+1}}+\varepsilon }$$

where α is the learning rate; $m_{n}$ and $v_{n}$ are the exponentially weighted moving average of the gradient and gradient squared for the nth iteration, respectively; and ε is a very small constant used to avoid the case where the divisor is zero.

An initial value of $D_{0}$ is chosen as the starting point of the iteration. It is also necessary to choose an appropriate learning rate α and two exponentially weighted moving average coefficients $\beta_{1}$ and $\beta_{2}$.

Starting from the initial value $D_{0}$, the next approximate solution $D_{1}$ is obtained via continuous iterative computation, and then $D_{1}$ is used as a new starting point to continue iterative computation to obtain the next approximate solution $D_{2}$, and so on, until the condition of stopping iteration is satisfied.

Specifically, the iterative calculation is given by the following:

(36) $$m_{n+1}=\beta_{1}\cdot m_{n}+(1-\beta_{1})\cdot f(D_{n})$$

(37) $$v_{n+1}=\beta_{2}\cdot v_{n}+(1-\beta_{2})\cdot f^{2}(D_{n})$$

(38) $$D_{n+1}=D_{n}-\alpha \cdot \frac{m_{n}}{\sqrt{v_{n+1}}+\varepsilon }$$

where $f(D_{n})$ is the derivative of the function f(D) at $D_{n}$.

Determine whether the absolute or relative error of the iteration sequence is less than the preset accuracy. If the iteration converges, stop the iteration and output the final approximate solution D; otherwise, continue the iterative computation.

The approximate solution D is the minimum safe spacing.

### 4.2. Simulation Calculation and Analysis

Conditional assumptions:

(1) It is assumed that only the horizontal safety spacing between aircraft A and aircraft B in programmed independent routes is studied;
(2) The position errors affecting the two aircraft are independent and joint;
(3) The flight paths of the two aircraft are straight lines.

This study uses the operational scenario at Zigong Airport in Sichuan Province to exemplify the lateral spacing during the fusion operation of unmanned and manned aircraft along their flight paths. To incorporate the impacts of collision avoidance equipment error, human factor error, meteorological condition error, and other factors, we conducted a thorough review of the relevant literature and considered the specific context of Zigong Airport. The factor values for the fusion operation of manned and unmanned aircraft are determined accordingly. Detailed data values and experimental results are presented in Table 4.

Given the fusion operation of unmanned aircraft and manned aircraft, the target safety level of lateral collision risk according to ICAO is taken as $TLS=1.0\times 10^{-7}$. We selected the Table 4 Zigong Airport fusion operation aircraft data as well as the environmental data to conduct the collision risk simulation experiment.

**Figure 10:**
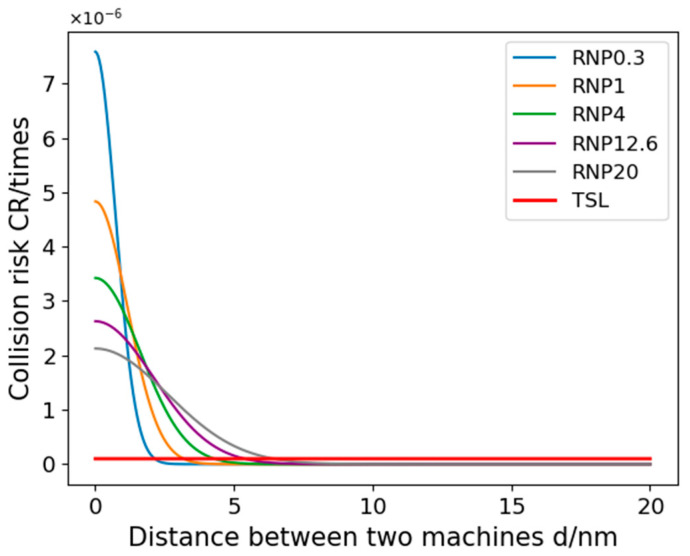
Variation in collision risk with lateral spacing between two airplanes for different RNP performances.

In Simulation Scheme 1, we altered only the RNP (Required Navigation Performance) and selected five different RNP levels for comparative analysis. To enhance clarity in the experimental results, this scheme excludes considerations of RCP (Required Communication Performance) and RSP (Required Surveillance Performance). Figure 10 illustrates the relationship between the collision risk and the minimum lateral safety distance between the two aircraft, with the determined safety distances detailed in Table 5.

The results of Experiment 1 show that the risk of collision between aircraft gradually decreases as the UAV spacing increases, which is consistent with the actual situation. The results in Figure 8 show that when fusion operations use different navigation performance (RNP), the effect on lateral safety spacing is significant, so special attention needs to be paid to the consideration of RNP performance. In addition, variations in RNP performance may also lead to an increase in collision risk at the same aircraft spacing. The calculated minimum safe spacing of aircraft for some RNP performances is listed in Table 5, and the difference between the five performances from RNP0.3 to RNP20 is more than 1 nautical mile, which also indicates that the size of the RNP performance has a large effect on the minimum safe spacing of aircraft. At the same time, other factors, such as weather conditions and communication equipment performance, need to be considered in the formulation of the minimum safe distance for aircraft. Therefore, in practical application, it is necessary to comprehensively consider a variety of factors in order to develop more scientific and reasonable aircraft spacing requirements to ensure the safe operation of aircraft.

**Figure 11:**
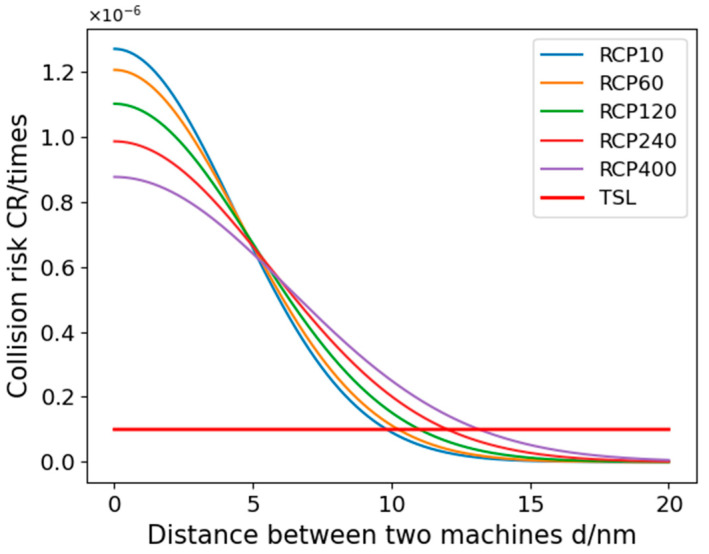
Variation in collision risk with lateral spacing between two airplanes for different RCP performances.

In Simulation Scheme 2, we focus solely on altering the RCP (Required Communication Performance). We have chosen five different RCP levels for a comparative study. To ensure clarity in the experimental outcomes, this scheme excludes RNP (Required Navigation Performance) and RSP (Required Surveillance Performance) considerations. Figure 11 depicts the relationship between collision risk and the minimum lateral safety distance between the two aircraft, with the calculated safe distances detailed in Table 6.

The results of Experiment 2 show that the inter-aircraft collision risk shows a gradual decrease as the spacing between UAVs and manned aircraft increases. In addition, an increase in the RCP parameter also leads to an increase in collision risk at the same aircraft spacing. The communication performance (RCP) parameters listed in Figure 9 differ from each other in order to see the specific gap, which shows that the communication performance has less influence on the collision risk of aircraft, which is consistent with the actual situation. The calculated minimum safe spacing between manned and unmanned aircraft under some of the RCP performance is listed in Table 6. From Table 6, it can be seen that the impact of RCP10 to RCP400 on the lateral minimum safe spacing is around 0.1 nautical miles, and these data are not negligible, which can provide a reference for the safe operation of the aircraft.

**Figure 12:**
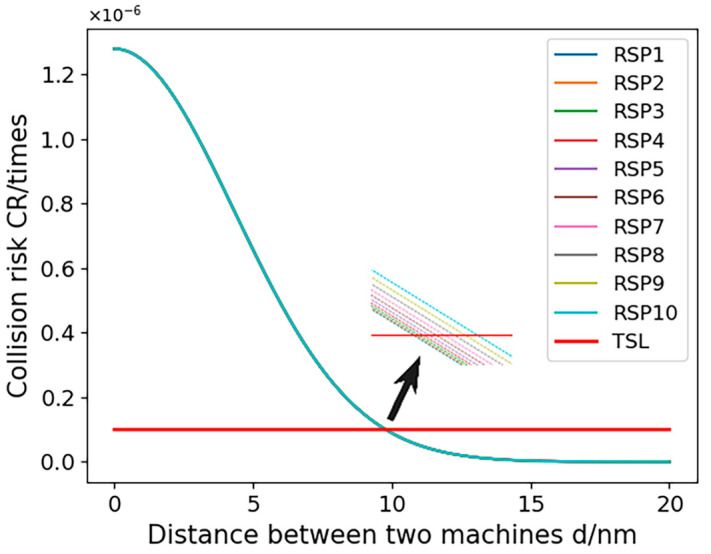
Variation in collision risk with lateral spacing between two airplanes for different RSP performances.

In Simulation Scheme 3, we exclusively focus on varying the RSP (Required Surveillance Performance), selecting 10 different RSP levels for comparative analysis. To enhance clarity in the results, this scheme omits considerations of RNP (Required Navigation Performance) and RCP. Figure 12 illustrates the correlation between collision risk and the minimum lateral safety distance between aircraft, with the determined safe distances presented in Table 7.

The results of Experiment 3 show that the risk of inter-aircraft collision gradually decreases as the UAV manned aircraft spacing increases. The calculated minimum safe spacing between manned and unmanned aircraft for some of the RSP performances is listed in Table 7. Analyzing the calculation results, the surveillance performance has a small impact on the collision risk of the aircraft, which is less than 0.002 nautical miles from RSP1 to RSP10. And the increase in the RSP parameter also increases the increase in collision risk under the same aircraft spacing, but the change is very small, and the pattern is consistent with the actual situation.

**Figure 13:**
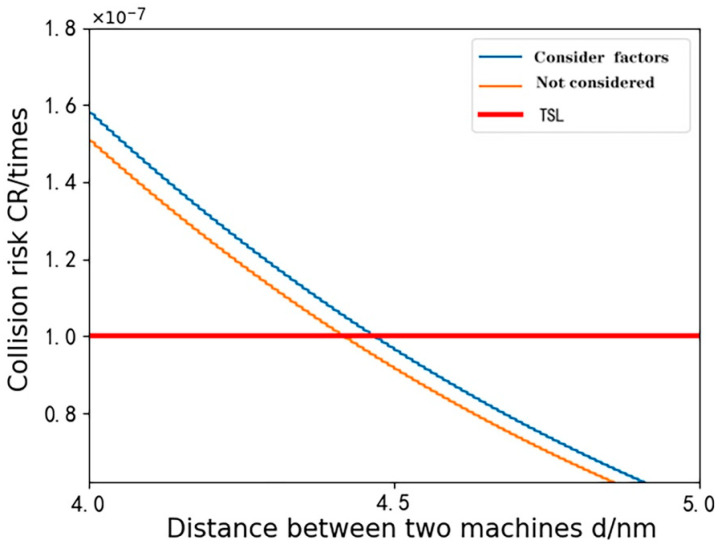
Crash risk versus the size of the two levels of lateral spacing.

In Simulation Scheme 4, the first scenario solely examines the impact of CNS (communication, navigation, and surveillance) performance. The second scenario expands the focus to include CNS performance, human factors, onboard collision avoidance systems, and meteorological conditions. This comparative approach uses performance parameters set as RNP1, RCP10, and RSP1. Figure 13 depicts the relationship between collision risk and the minimum lateral safety spacing between aircraft, with the calculated safe distances detailed in Table 8.

The results of Experiment 4 show that there is a relationship between the distance between the two aircraft and the risk of collision when manned and unmanned aircraft are fused in operation. As the distance between them increases, the probability of collision gradually decreases. It is worth noting that from Figure 11, we also observe that the intersection of the collision risk curve with TLS (target safety level) is delayed, and the minimum safe spacing $D_{\min}$ of the aircraft increases after considering other influencing factors. The calculated minimum safety distance in Table 8 increases from the previous 4.42 n mile to 4.47 n mile. Combining the analyses of Experiments 1, 2, 3, and 4, the CNS (communication, navigation, and surveillance) performance has a significant effect on the safety distance of the aircraft, although its role varies among the experiments and has a significant effect. The results of Experiment 4 also showed that in addition to CNS performance, factors such as human factors, onboard equipment, and meteorological conditions also have an effect on safe spacing. This study provides insights into understanding the relationship between inter-aircraft collision risk and safety spacing. Taking other factors into account makes the requirements for safety spacing more scientific and reasonable and helps to improve the overall safety of aircraft fusion operations.

## 5. Conclusions

When the collision count aligns with the target safety level, the derived spacing between two aircraft becomes the minimum lateral safety spacing. Higher positioning accuracy leads to reduced minimum safety spacing. Utilizing this methodology, further studies can investigate the minimum vertical and longitudinal spacing for fusion operations involving manned and unmanned aircraft. The experimental data confirm that increased accuracy results in smaller safety spacings, consistent with standard aircraft operating procedures. These findings also validate the 10 km horizontal safety spacing between manned and unmanned aircraft, as specified by Zigong General Aviation Airport.

This study integrates the impacts of CNS positioning error, human factors, onboard equipment, and meteorological conditions into the classical collision risk model, forming the UM-EVENT model. This model, applied to the fusion operation scenario, allows for calculating the minimum lateral safety spacing. The simulation results demonstrate the practicality and applicability of this method in determining safety spacings for UAV-manned aircraft fusion operations. The UM-EVENT model calculates the minimum lateral spacing for fusion operations based on specified CNS performance parameters and target level of safety (TLS). It also evaluates the collision risk associated with this spacing. Thus, it offers theoretical support for the fusion operation of UAVs and manned aircraft and contributes to the integration of UAVs into the national airspace system.

## 6. Outlook

The research on the safety spacing in the integrated operation of manned and unmanned aircraft is still in the experimental stage. Due to constraints such as time, experimental environment, equipment, team resources, and individual capabilities, this paper lacks a perfect error margin. Accordingly, the following points are proposed for in-depth investigation:

(1) Refined Error Analysis: Undertake an in-depth study for a more nuanced error analysis, considering factors such as time, experimental environment, and equipment. This aims to quantify sources of error, thereby enhancing the credibility of research outcomes.
(2) Field Verification and Validation: Conduct on-site validation of research outcomes utilizing real operational data to verify the applicability of experimental results. Additionally, collaboration with other experimental teams for multi-center validation can enhance the generalizability and reproducibility of the study.
(3) Risk Assessment and Emergency Strategies: Conduct extensive research and development on risk assessment models, taking into account various risk scenarios. Propose corresponding emergency strategies to establish comprehensive and operational safety spacing standards, thereby augmenting the overall safety of integrated manned and unmanned aircraft operations.

## Figures and Tables

**Table 1:** RNP performance parameters.

Data	Accuracy (Nautical Miles)
RNP0.3	±0.3
RNP1	±1
RNP4	±4
RNP12.6	±12.6
RNP20	±20

**Table 2:** RCP performance parameters.

Data	Processing Time	Contiguity	Available	Completeness
RCP10	10 s	0.999	0.99998	$10^{-5}$
RCP60	60 s	0.999	0.9999	$10^{-5}$
RCP120	120 s	0.999	0.9999	$10^{-5}$
RCP240	240 s	0.999	0.999	$10^{-5}$
RCP400	400 s	0.999	0.999	$10^{-5}$

**Table 3:** RSP performance parameters.

Data	Specified Airspace	Refresh Rate	Reaction Time
RSP1	route	≤1 s	2 s
RSP2	terminal area	≤2 s	2 s
RSP3	run the scene	≤3 s	2 s
RSP4	parallel entry	≤4 s	2 s

**Table 4:** Other factors affecting the positioning error of the aircraft take values.

Influencing Factor	Twin-Tailed Scorpion Drone	Cessna 172
Actual heading angle $\theta_{a}$	45°	225°
Theoretical heading angle $\theta_{t}$	47°	226°
Human cognitive reliability $p_{n}$	80%	90%
Pressure P	700 hPa	700 hPa
Temp T	10 °C	10 °C
Relative humidity H	50%	50%
Wavelength of light λ	550 nm	600 nm
Transmitter Antenna Gain $G_{t}$	10 dBi	15 dBi
Transmitter output power $P_{W}$	20 dBm	30 dBm
Receiving Antenna Gain $G_{r}$	12 dBi	13 dBi
Signal Transmission Distance $d_{s}$	5 km	7 km
Aircraft drag coefficient $C_{d}$	0.04	0.03
Atmospheric density ρ	0.85 kg/m^3^	0.85 kg/m^3^
Speed of aircraft V	200 km/h	180 km/h
Reference area of aircraft S	20 m^2^	18 m^2^
Air velocity $V_{W}$	15 m/s	10 m/s
Angle between wind and heading α	30°	30°

**Table 5:** Lateral safety spacing for different RNP performance.

RNP Performance	Minimum Lateral Safety Distance (n Mile)
RNP0.3	2.146
RNP1	3.193
RNP4	4.299
RNP12.6	5.385
RNP20	6.442

**Table 6:** Lateral safety spacing for different RCP performances.

RCP Performance	Minimum Lateral Safety Distance (n Mile)
RCP10	9.776
RCP60	9.829
RCP120	9.990
RCP240	10.594
RCP400	11.839

**Table 7:** Lateral safety spacing for different RSP performances.

RSP Performance	Minimum Lateral Safety Distance (n Mile)
RSP1	9.776883
RSP2	9.776930
RSP3	9.777007
RSP4	9.777113
RSP5	9.777251
RSP6	9.777419
RSP7	9.777614
RSP8	9.777842
RSP9	9.778101
RSP10	9.778390

**Table 8:** Minimum lateral safety spacing corresponding to the combined factors.

Influencing Factors	$\mathrm{Minimum}\;\mathrm{Lateral}\;\mathrm{Safety}\;\mathrm{Distance}\;D_{\min}$
Consider only CNS performance	4.42 n mile
Consideration of Synthesis Factors in UM-Event Modeling	4.47 n mile

## Data Availability

The data that support the findings of this research are available from the author, C.H., upon reasonable request.
